
JOURNAL INFORMATION
==============================
NLM Title Abbreviation: Curr Med Sci
Journal ID: Curr Med Sci
NLM Journal ID: 101729993

Current Medical Science

ISSN: 2096-5230
EISSN: 2523-899X

ARTICLE INFORMATION
==============================
PMCID: PMC7902558
PMID: 32681266
DOI: 10.1007/s11596-020-2219-8
Article ID: 2219
Article version: 1
Subjects: Erratum

Erratum to: The Role of CARD9 in Metabolic Diseases

Tian Cheng czgg@hust.edu.cn

Tuo Ya-li
Lu Yi
Xu Chuan-rui
Xiang Ming xiangming@tjmu.edu.cn

grid.33199.31 0000 0004 0368 7223 Department of Pharmacology, School of Pharmacy, Tongji Medical College, Huazhong University of Science and Technology, Wuhan, 430030 China
Electronic publication date: 2020 Jul 17
Print publication date: 2020
Volume: 40
Issue: 3
First page: 595
Last page: 595
Copyright: © The Author(s) 2020
License: Open Access This article is licensed under a Creative Commons Attribution 4.0 International License https://creativecommons.org/licenses/by/4.0/), which permits use, sharing, adaptation, distribution and reproduction in any medium or format, as long as you give appropriate credit to the original author(s) and the source, provide a link to the Creative Commons license, and indicate if changes were made.
License URL: https://creativecommons.org/licenses/by/4.0/

Erratum for: The Role of CARD9 in Metabolic Diseases. 40 2 26 4 2020 199 204 Curr Med Sci 10.1007/s11596-020-2166-4 32337681
issue-copyright-statement: © Huazhong University of Science and Technology 2020

==============================
The online version of the original article can be found at 10.1007/s11596-020-2166-4

